# Environmental aspects of health care in the Grampian NHS region and the place of telehealth

**DOI:** 10.1258/jtt.2010.004015

**Published:** 2010-06

**Authors:** Richard Wootton, Alex Tait, Amanda Croft

**Affiliations:** * Scottish Centre for Telehealth, Aberdeen; † Estates Department, Elgin; ‡ Ashgrove House, Aberdeen Royal Infirmary, Aberdeen, UK

## Abstract

Detailed information about the composition of the carbon footprint of the NHS in the Grampian health region, and in Scotland generally, is not available at present. Based on the limited information available, our best guess is that travel emissions in Grampian are substantial, perhaps 49,000 tonnes CO_2_ per year. This is equivalent to 233 million km of car travel per year. A well-established telemedicine network in the Grampian region, which saves over 2000 patient journeys a year from community hospitals, avoids about 260,000 km travel per year, or about 59 tonnes CO_2_ per year. Therefore using telehealth as it has been used historically (primarily to facilitate hospital-to-hospital interactions) seems unlikely to have a major environmental impact – although of course there may be other good reasons for persevering with conventional telehealth. On the other hand, telehealth might be useful in reducing staff travel and to a lesser extent, visitor travel. It looks particularly promising for reducing outpatient travel, where substantial carbon savings might be made by reconfiguring the way that certain services are provided.

## Introduction

Until recently there has been little readily-available information about the composition of the carbon footprint of the NHS in Scotland. It is therefore difficult to know what influence telehealth presently has, or might have in future. Such information is a pre-requisite for any kind of planning work about how telehealth might be developed for NHS purposes.

Detailed information about the carbon emissions resulting from NHS work in England was published in 2008.^1^ We have used this information to extrapolate to the case of Scotland. A recently published report also provides estimates of the carbon footprint of NHS Scotland.^2^ The two estimates combined can be used as a ‘best guess’ at the true figure.

Using the best-guess figure for NHS Scotland allows the travel emissions from the Grampian health region to be estimated. This region contains some well-established telehealth applications, and the resultant carbon savings can be calculated fairly accurately. These telehealth carbon savings can then be viewed in the context of the emissions from the Grampian region as a whole.

## The NHS and the environment

**Figure 1:**
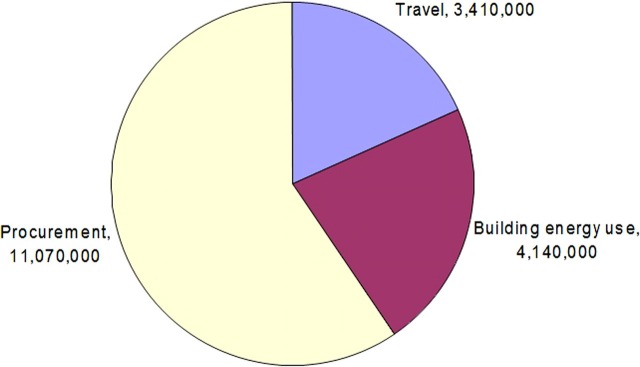
NHS (England) – the total CO_2_ emissions were 18.6 million tonnes in 2004^1^

The NHS is a large organization and consequently it has a substantial impact on the environment. There are three sources of carbon emissions that make up the carbon footprint of the NHS.^1^ The largest source is the ‘embodied’ carbon emissions, i.e. those resulting from the goods and services consumed by the NHS. Second, there are the direct carbon emissions associated with buildings, and finally there are the carbon emissions resulting from travel connected with NHS activities. In England, roughly half of the CO_2_ emitted comes from the first source, called procurement. About a quarter each comes from building energy use and from travel (see Figure 1).

## Carbon reduction to date

Although procurement represents the largest component of the carbon footprint, efforts to reduce it are only just being made.^3^ It remains to be seen what can actually be achieved in practice.

On the other hand, the NHS has made continual attempts to reduce energy consumption. Since 1985, the target has been a 2% reduction in energy consumption per year. This has been achieved by switching from the use of coal and oil as energy sources to natural gas, by installing more modern plants and by improving the energy efficiency of buildings (e.g. better insulation and lighting). These efforts have been successful, and since 1985 the overall energy use – and thus the CO_2_ emitted – has been reduced by approximately 40%.^4^ Note, however, that it will not be possible to continue the energy reduction indefinitely, as the law of diminishing returns applies.

## Travel

### Travel emissions in England

**Figure 2:**
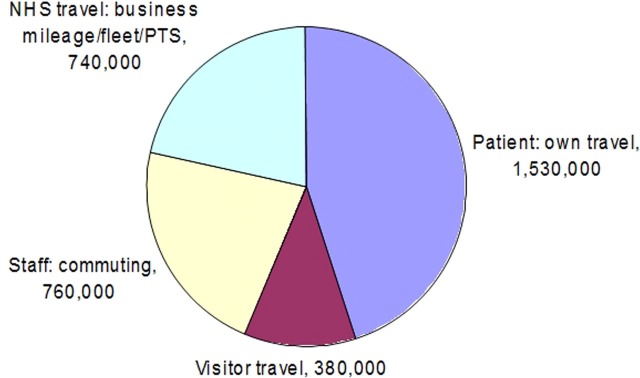
NHS (England) – the total CO_2_ emissions due to travel were 3.4 million tonnes in 2004^1^

The final component of the carbon footprint is due to travel. Here, roughly half of the CO_2_ emitted is due to staff, either commuting to work or conducting business-related travel (see Figure 2). Most of the rest is due to the patients, although about 10% of the total is due to visitors.^1^

### Travel emissions in Scotland

At the time the present study began (2008), information on NHS travel emissions in Scotland was not readily available, and we therefore estimated it from the case of England. The building energy emissions of the NHS in Scotland are about 10% of those of the NHS in England.^4^ Suppose that NHS travel in Scotland is in the same proportions as the NHS in England, then the total travel emissions would amount to 337,706 tonnes per year (estimate A). By analogy with England, the breakdown would be as shown in Table 1. Note that the average population density in Scotland is much lower than in England (65 /km^2^ vs 383 /km^2^),^5^ which may affect these estimates.

**Table 1:** Estimated annual travel emissions from NHS Scotland. Estimate A is from the present paper (see text); Estimate B is from the recent Health Facilities Scotland report.^2^ The best-guess value is the mean of the two estimates

Component	Estimate A Tonnes CO_2_	Estimate B Tonnes CO_2_	Best guess Tonnes CO_2_
Patient own	151,522	260,000	206,000
Staff – commuting	75,266	70,000	73,000
Staff – business	73,285	150,000	112,000
Visitor	37,633	160,000	99,000
*Total*	*337,706*	*640,000*	*489,000*

Since these estimates were made, NHS Scotland has commissioned a carbon footprint report from external consultants.^2^ The overall travel emission (estimate B) is approximately twice as high as our own (see Table 1). The Health Facilities Scotland report states that its values are likely to contain considerable uncertainties because of the way they were derived. For the purposes of the subsequent discussion here, we will use the mean of the two estimates as the best guess at the true carbon emissions of NHS Scotland.

### Travel emissions in the Grampian region

There is little published information about NHS travel in the Grampian region. Carbon emissions can be crudely estimated from the NHS Scotland figure derived above. The Grampian region contains approximately 10% of the Scottish population. If NHS travel is in proportion, then the breakdown for the Grampian region would be as shown in Table 2.

**Table 2:** Estimated annual travel emissions from the NHS Grampian region and the equivalent car-travel distance (assuming an average petrol car^6^)

Component	Tonnes CO_2_	Equivalent car-travel distance (km)
Patient own	20,576	98 million
Staff – commuting	7,263	35 million
Staff – business	11,164	53 million
Visitor	9,882	47 million
*Total*	*48,885*	*233 million*

## Telehealth

What part might be played by telehealth in reducing the carbon footprint of the NHS? Telehealth is the provision of health care at a distance. Thus a procedure involving telehealth will mean that travel is reduced or avoided – that is, travel on the part of the health-care worker, the patient or others. In principle, therefore, the use of telehealth is to be encouraged on environmental grounds (leaving aside awkward questions about resource allocation).

## Examples of telehealth

Conventionally, telehealth has been used to improve the efficiency of health-care delivery. Most of the experience to date has been in the secondary or tertiary health sectors, where telehealth is often used to improve access to specialist opinions. This saves travel on the part of the patient or doctor. Examples of telehealth being used to reduce patient travel in the north east of Scotland, from which environmental savings can be calculated, include the Grampian minor injuries service and the specialist referral service for patients in the Shetland Islands.

### Minor injuries

In the north east of Scotland, telehealth is used as a decision-support aid at units treating patients with minor injuries. These units are usually based in small, community hospitals and staffed by nurses rather than doctors. Obviously patients with significant injuries are not treated locally, but transferred to a larger hospital. Many of the minor injuries can be managed locally, by nurses working to pre-agreed protocols. In a proportion of cases, where the best course of action is not immediately clear, the nurse will request advice from doctors at the main centre, the Aberdeen Royal Infirmary.

Using a video link allows the doctor providing telehealth advice to see the patient and provide appropriate advice. In some 90–95% of these requests for telehealth advice, the result is that the patient does not need to be transferred to Aberdeen.^7^ Since relatively large numbers of patients are managed using telehealth, the avoided travel is substantial. There are, of course, other benefits, such as the convenience to patients.

During 2007 there were 2061 teleconsultations between the 14 minor injury units in the Grampian region and the main centre at the Aberdeen Royal Infirmary. This equates to avoided travel of some 260,000 km, or a saving of 55 tonnes of CO_2_ per annum (see Table 3).

**Table 3:** The total travel savings due to minor injuries telemedicine in the Grampian region during 2007 amounted to approximately 260,000 km of road travel. There were 2061 teleconsultations between the 14 minor injury units and the main centre at the Aberdeen Royal Infirmary. There were 443 teleconsultations from Banff. Assuming that 95% of the teleconsultations resulted in an avoided journey to Aberdeen, then in the case of the Banff centre the total travel distance saved was 75,753 km. If the travel had been by car, the CO_2_ emission (0.21 kg/km, i.e. an average petrol car^6^) would have amounted to 15.9 tonnes

Unit	Distance to ARI (km)	Cases during 2007	Avoided travel (km)	Avoided CO_2_ (tonnes)
Banff	90	443	75,753	15.9
Fraserburgh	67	455	57,922	12.2
Huntly	60	117	13,338	2.8
Inverurie	24	24	1094	0.2
Peterhead	52	377	37,248	7.8
Turiff	53	319	32,123	6.8
Other	75*	326	46,455	9.8
*Total*		*2061*	*258,978*	*55.4*

*The average distance of the other 8 units

### Head and neck cancer

A more recent telehealth service allows patients in the Shetland Islands with suspected head and neck cancer to be assessed by a specialist without needing to make a journey to the Aberdeen Royal Infirmary. Use of a video link allows the specialist to decide whether local management is possible, or whether the patient needs to be referred to Aberdeen for treatment.

During 2007/08 there were 42 such teleconsultations, as a result of which 42 patient journeys were avoided. The trip from Shetland to Aberdeen requires two road journeys and a flight, resulting in an emission of 61 kg CO_2_. The avoided travel amounts to a saving of 3.7 tonnes of CO_2_ per annum.^8^

## Telehealth in context

As shown above, the total travel emissions from the NHS in Grampian can be estimated to be about 49,000 tonnes of CO_2_ per year. The total savings in avoided travel from the two telehealth examples cited above amount to 59 tonnes of CO_2_, which is approximately 0.1% of the travel emissions in Grampian. This is not to imply that these savings are not worthwhile. Simply, that in relation to the overall carbon footprint of NHS Grampian, these two examples of telehealth are fairly insignificant.

What are the carbon savings resulting from all telehealth work currently being conducted in the Grampian region? At present, this is impossible to know since there is a dearth of quantitative data. However, even if there was ten times as much telehealth going on as the two examples quoted above (which seems unlikely), the avoided CO_2_ would still only amount to 590 tonnes. It seems safe to conclude that telehealth, when used conventionally, is not likely to have a big influence on the environmental impact of the NHS.

## Potential of telehealth

Can telehealth therefore be used in a different way? What is the scope for reducing travel emissions in the NHS? Obviously one strategy would be to improve the efficiency of travel generally, i.e. to provide better and more effective public transport. However, this is not really under the control of the health sector to any significant degree.

Other strategies specific to the NHS include:

1. Reducing staff travel, e.g. by the use of video meetings or by teleworking;
2. Reducing patient travel, e.g. by holding outreach clinics instead of hospital outpatient clinics;
3. Reducing visitor journeys to hospital, e.g. by use of ‘video visiting’ for relatives.

Staff travel and patient travel are the two big-ticket items, collectively responsible for about 80% of the travel emissions. Efforts should therefore be concentrated on them.

### Staff travel

Teleworking and video meetings are used to some extent in NHS Scotland by staff for whom it is appropriate. Information about overall levels of usage is not presently collected. However, video linking is being promoted and improved facilities are being provided at a number of locations in the NHS Grampian region, for example.

More detailed information about travel associated with the patient travel scheme (PTS) and the ambulance service would be helpful in formulating suggestions for the future use of telehealth in Scotland.

### Patient travel

Patient travel comprises that associated with primary care, e.g. visits to the general practitioner (GP), and with secondary/tertiary care, e.g. visits to hospital, either as an inpatient or an outpatient. In the Grampian region, the emissions resulting from all patient travel is estimated to be about 21,000 tonnes CO_2_ per year (Table 2).

What is the composition of ‘patient-own travel’? For example, how much hospital travel represents travel for inpatient treatment and how much is travel to outpatient clinics? This information does not appear to be separately recorded for Scotland or for England at present. However, it is safe to assume that outpatient travel represents a substantial proportion of patient-own travel.

In the Grampian region, total patient travel for hospital outpatient appointments amounts to 17.6 million km per year.^9^ This corresponds to the emission of approximately 3700 tonnes of CO_2_ (using 0.21 kg/km). That amounts to about 20% of the estimated patient travel in Table 2. This implies that patient travel for inpatient and for primary care purposes is some four times larger than for outpatient appointments. Clearly more information is required before the plausibility of these figures can be judged.

Whatever the relative magnitudes of the patient travel components turn out to be, there is a compelling case that outpatient travel could be reduced by employing telehealth. Evidence from other countries shows that a substantial proportion of outpatient travel can be avoided by use of telehealth.^10^ In one Finnish study, over half of the teleconsultation patients could be treated by the GP and did not need to go to hospital, following telehealth advice from the consultant.^11^ In another Finnish study, almost all (98%) of general surgery patients were able to avoid a journey to hospital by using a telehealth link at their GP's premises.^12^ Table 4 provides a summary of recent studies reporting avoided travel to hospital (e.g. for outpatient visits) as a result of telemedicine of various kinds.

**Table 4:** Studies reporting avoided travel to hospital (e.g. for outpatient visits) as a result of telemedicine

	Specialty	Referrers	No. of patients	Duration of study	Modality	Avoided hospital visits, e.g. to the outpatient clinic
Molinari, 2002^13^	Cardiology	GPs	456	1 month	Telephone and telephone-transmitted ECG	63% of the 134 patients suggested for hospitalization by the GP
Granlund, 2003^14^	Dermatology	GPs	23 videoconferencing, 25 face-to-face	12 months	Videoconferencing	68% of face-to-face group needed to go to hospital, but only 41% of the videoconferencing group
Lamminen, 2000^15^	Dermatology	GPs	25	8 months	Videoconferencing	72%
Made, 1999^16^	ENT	GPs	32	21 months	Videoconferencing	39%
Bowater, 2001^17^	General	Remote GP	90	24 months	Videoconferencing	75%
Harno, 2000^18^	General	GPs	292	8 months	Intranet store-and-forward system	43% of the intranet referrals resulted in outpatient visits, compared with 79% in conventional referral group
Chan, 2001^19^	Geriatrics	Nursing home staff	198	12 months	Videoconferencing	89% of nursing home visits avoided
Hui, 2002^20^	Geriatrics	Nursing home staff	(1001 teleconsultations in seven disciplines)	12 months	Videoconferencing	9% reduction in hospital emergency department attendances; 11% reduction in bed-days at hospital
Harno, 1999^21^	Internal medicine and surgery	GPs	c30,000 referrals	12 months	Electronic referral system	Over 95% of paper referrals in the conventional system led to an outpatient visit, whereas only one-third of the teleconsultations resulted in actual outpatient visits
Paiva, 2001^22^	Neurology	GPs	90	13 months	Videoconferencing	46%
Lamminen, 1999^23^	Ophthalmology	GPs	24	10 months	Videoconferencing	71%
Hanson, 2008^24^	Ophthalmology	Optometrists	171	24 months	Store-and-forward web-based system	48%
Fortin, 2003^25^	Orthopaedics and radiology	GPs at a local hospital	118	13 months	Videoconferencing and store-and-forward	20%
Trott, 1998^26^	Psychiatry	Local hospital	240	6 months	Videoconferencing	40%
Worth, 2003^27^	Psychiatry	GP	303 intended referrals (595 in all)	12 months	Phone and email	24%
Aarnio, 2000^12^	Surgery	GPs	50	-	Videoconferencing	98%
Jaatinen, 2002^11^	Surgery, geriatrics	GPs	93	5 months	Web-based store-and-forward system	48% of tele-referral group (n = 23) avoided hospital treatment
Johnson, 1998^28^	Ultrasound	Local hospital	146	?	Store-and-forward and videoconferencing	42%

There is also emerging evidence for telephone follow-up in other areas, for example rheumatology,^29^ orthopaedics^30^ and hand surgery^31^

What is the potential in Scotland? Suppose that 20% of outpatient visits in the Grampian region could be replaced by local telehealth, then the potential CO_2_ saving would be 704 tonnes. This is 12 times as much as the two telehealth examples quoted above.

### Visitor travel

Visitor travel is presently thought to be a minor component of the overall travel emissions, so it probably does not deserve a great deal of attention. On the other hand, visitor travel might be susceptible to reductions through telehealth. This would have benefits in terms of reduced CO_2_ emissions, to say nothing of reduced demand for car parking at the major hospital sites. Unfortunately, there is a dearth of factual information about visitor travel, whether in England, in Scotland or in the Grampian health region, so it is impossible to estimate costs and benefits at present.

## Conclusion

Obtaining accurate patient and visitor travel information would greatly enhance our understanding of the true carbon footprint of the NHS in Scotland. If detailed information about staff travel could also be obtained then a much better overall picture would result; this would allow rational planning about strategies for reductions. Accurate information is needed about all four components of travel:

1. NHS business travel, including the patient travel scheme;
2. Staff commuting;
3. Patient (outpatient and inpatient) travel;
4. Visitor travel.

This could be obtained by survey.

It would also be helpful to conduct some pilot work to establish the feasibility of peripheral outpatient clinics in certain specialties, i.e. outreach clinics supported by telehealth. This proposed work on travel reduction would build on earlier studies.

Using telehealth as it has been used historically (primarily to facilitate hospital-to-hospital interactions) seems unlikely to have a major environmental impact – although of course there may be other good reasons for persevering with conventional telehealth. On the other hand, telehealth might be useful in reducing staff travel and to a lesser extent, visitor travel. In the context of the NHS in Scotland, it looks particularly promising for reducing outpatient travel, where substantial carbon savings might be made by reconfiguring the way that certain services are provided.

The principal advantage of telehealth is in facilitating equitable access, so perhaps its main role is in providing services to disadvantaged peoples. The main environmental benefits lie in the form of avoided travel.
